# Fulminant type 1 diabetes mellitus associated with Coxsackievirus type B1 infection during pregnancy: a case report

**DOI:** 10.1186/s13256-019-2130-8

**Published:** 2019-06-19

**Authors:** Takahiro Hayakawa, Yoshio Nakano, Kana Hayakawa, Hiroaki Yoshimatu, Yoshikazu Hori, Kazuki Yamanishi, Hirofumi Yamanishi, Takayuki Ota, Tokuzo Fujimoto

**Affiliations:** Department of Internal Medicine, Kinan Hospital, 46-70, Shinjo, Tanabe City, Wakayama 646-8588 Japan

**Keywords:** Fulminant type 1 diabetes mellitus, Coxsackievirus B1, Pregnancy, Antiviral antibody

## Abstract

**Background:**

Fulminant type 1 diabetes is characterized by an intrinsic insulin deficiency resulting from the severe destruction of pancreatic β cells and it rapidly leads to ketoacidosis. However, the association between fulminant type 1 diabetes in pregnancy and specific viral infections has not been reported.

**Case presentation:**

The patient in this study was a 31-year-old Japanese woman, and at 30 weeks of pregnancy she was admitted with marked fatigue. Fetal bradycardia was noted, and the child was delivered by emergency cesarean section but was stillborn. The maternal blood sugar level was high (427 mg/dL), but the glycated hemoglobin value was 6.2%; therefore, fulminant type 1 diabetes was suspected. Serum antibody testing confirmed a Coxsackievirus B1 infection. The patient in this case had fulminant type 1 diabetes in pregnancy associated with Coxsackievirus B1.

**Conclusion:**

This case highlights that fulminant type 1 diabetes in pregnancy may be associated with Coxsackievirus B1 infection.

## Introduction

Fulminant type 1 diabetes (FT1D) is a subtype of type 1 diabetes (T1D); it is characterized by the abrupt onset of insulin-deficient hyperglycemia and ketoacidosis within a few days [1].

The specific etiology and pathogenesis of FT1D are unclear and may be related to genetic predisposition, autoimmunity, viral infection, and pregnancy.

Viral infection is strongly associated with fulminant diabetes, as reported previously, and can be associated with frequent flu-like symptoms along with a seasonal variation in time of onset. A viral infection is suggested to play a role in rapid β cell destruction.

Epidemiological studies have indicated an association between enteroviruses (EVs) and T1D [2]. Certain class II human leukocyte antigens (HLA) are known to increase susceptibility to FT1D during pregnancy [3]. However, the association between FT1D in pregnancy and specific viral infections has not been reported.

We report the case of a patient who developed FT1D in pregnancy accompanied by Coxsackievirus B1 infection.

## Case presentation

Our patient was a 31-year-old Japanese woman, gravida 1, para 1. She had no remarkable medical history, and there were no abnormalities during her medical examination. Her father and uncle had type 2 diabetes requiring insulin therapy. However, her sister did not have diabetes. The weight gain during her first 3 months of pregnancy was 1.1 kg. A regular examination at 29 weeks and 5 days was normal; her blood glucose level was 73 mg/dL, with negative levels of urinary glucose, and 3+ urinary ketone bodies.

At 30 weeks and 6 days, she was admitted with marked fatigue and dyspnea. However, she did not present with fever, headache, costochondritis, pharyngitis, diarrhea, maculopapular non-pruritic rash, nausea, or flu-like symptoms such as a sore throat, cough, or rhinorrhea.

On admission, she was alert. A physical examination revealed the following: temperature (T), 36.7 °C; pulse rate (P), 94 beats per minute (bpm); respiratory rate (R), 18/minute; and blood pressure (BP), 121/65 mmHg; fetal bradycardia was also observed, for which emergency cesarean section was performed, but the child was stillborn. The laboratory findings revealed diabetic ketoacidosis (DKA), with random sample glucose of 427 mg/dL, C-peptide reactivity (CPR) of 0.04 ng/mL, arterial pH of 6.91, bicarbonate of 6.7 mEq/L, and 3+ urinary ketone bodies. Despite the presence of DKA, the glycated hemoglobin (HbA1c) value was within the normal range (6.2%), and urinary CPR was extremely low at 0.5 μg/day. In addition, the serum elastase-1 and lipase levels were increased to 1939 (< 300) ng/dL and 119 (11–53) U/L, respectively. Antibody level against glutamic acid decarboxylase was 1.2 (< 1.5) U/mL. Neither anti-insulin nor anti-insulinoma-associated antigen-2 antibodies were detected. HLA II haplotypes showed *DR8,12/DQ7,4*. These results were indicative of a FT1D diagnosis (Table 1).

**Table 1:** Laboratory data on admission

Hematology				ABGA (O_2_ 5 L/m)	
WBC	14,400 /μl	CRP	6.4 mg/dl	pH	6.91
neu.	74%	Amylase	139 IU/L	pCO_2_	33.7 mmHg
lym.	22%	Elastase1	1939 ng/dl	PO_2_	184 mmHg
mon.	3%	Lipase	119 U/L	HCO_3_^−^	6.5 mmol/L
eosino.	1%	Na	129 mEq/l	BE	−26.1 mmol/L
RBC	468 × 10^4^ /μl	K	6.7 mEq/l		
Hb	13.5 g/dl	Cl	95 mEq/l	Urine analysis	
Ht	40.7%	Glu	427 mg/dl	pH	5.0
Plt	47.8 × 10^4^ /μl	HbA1c	6.2%	Protein	(2+)
		Serum C-peptide	0.04 ng/ml	Glucose	(4+)
Chemistry		TSH	2.14 μIU/ml	Blood	(1+)
AST	14 IU/L	F-T4	1.03 ng/ml	Ketone	(4+)
ALT	12 IU/L			Urine C-peptide	0.5 μg/day
γ-GTP	12 IU/L				
T-Bil	0.5 mg/dl	Coagulation		Autoantibodies	
ALP	328 IU/l	PT	12.3 sec	GAD Ab	< 5.0 U/ml
TP	9 mg/dl	APTT	30.9 sec	IA-2 Ab	(−)
Alb	4.2 g/dl	FDP	8.47 μg/ml	Insulin Ab	< 0.4 U/ml
CK	29 IU/l	D-dimer	5.48 μg/ml		
CRE	0.87 mg/dl			HLA haplotype	
BUN	22 mg/dl			DR8, DR12, DQ7, DQ4	

*Ab* antibody, *ABGA* arterial blood gas analysis, *Alb* albumin**,** *ALP* alkaline phosphatase, *ALT* alanine aminotransferase, *APTT* activated partial thromboplastin time, *AST* aspartate aminotransferase, *BE* base excess, *BUN* blood urea nitrogen, *CK* creatine kinase, *CRE* creatinine, *CRP* C-reactive protein, *eosino*. eosinophils, *FDP* fibrin degradation product, *F-T4* free thyroxine, *GAD* glutamic acid decarboxylase, *γ-GTP* gamma-glutamyl transpeptidase, *GLU* glucose, *Hb* hemoglobin, *HbA1c* glycated hemoglobin, *HCO*_*3*_^−^ bicarbonate, *HLA* human leukocyte antigens_,_ *Ht* hematocrit, *lym*. lymphocytes, *mon*. monocytes, *neu*. neutrophils, *pCO*_*2*_ partial pressure of carbon dioxide, *pO*_*2*_ partial pressure of oxygen, *Plt* platelets, *PT* prothrombin time, *RBC* red blood cells, *T-Bil* total bilirubin, *TP* total protein, *TSH* thyroid-stimulating hormone, *WBC* white blood cells

She was diagnosed as having DKA due to FT1D. She was simultaneously treated with fluid replacement and continuous insulin infusion to maintain vital signs, plasma glucose, and electrolyte levels. On day 2, ketonuria, electrolytes, and vital signs had normalized. On day 3, the continuous infusion of insulin was withdrawn, and daily multiple insulin injections were administered with blood glucose monitoring. On day 24, she continued to undergo treatment with multiple daily insulin injection therapy with insulin aspart (25 U/day) and insulin degludec (12 U/day).

### Paired serum antiviral antibody test

To investigate the association between viral infection and FT1D, we performed serological testing for several viruses such as parainfluenza virus 1–3, Coxsackievirus A2–7, 9, 10, 16, B1–6, cytomegalovirus (CMV), Epstein–Barr virus (EBV), and human herpes virus (HHV) 6 at day 3, day 17, and day 38 (Table 2). A Coxsackievirus B1 antibody titer was significantly elevated from 1:32 to 1:256 (eightfold increase), whereas the other antibodies were not altered.

**Table 2:** Viral antibodies

	Day 3	Day 17	Day 38		Day 3	Day 17
Parainfluenza1	< 10	< 10		Coxsackie B3	< 4	< 4
Parainfluenza2	< 10	< 10		Coxsackie B4	< 4	< 4
Parainfluenza3	160	160		Coxsackie B5	< 4	< 4
Coxsackie A2	< 4	< 4		Coxsackie B6	< 4	< 4
Coxsackie A3	8	8		CMV IgM	0.49	0.79
Coxsackie A4	16	16		CMV IgG	< 2	< 2
Coxsackie A5	< 4	< 4		EBV anti-VCA IgM	0	0.1
Coxsackie A6	< 4	< 4		EBV anti-VCA IgG	2.2	2.6
Coxsackie A7	8	4		EBV anti-EBNA IgG	3.1	3.3
Coxsackie A9	4	8		HHV-6 IgM	< 10	< 10
Coxsackie A10	< 4	< 4		HHV-6 IgG	160	160
Coxsackie A16	< 4	< 16		HHV-7 IgM	< 10	< 10
Coxsackie B1	32	64	256	HHV-7 IgG	40	40
Coxsackie B2	< 4	< 4				

*CMV* cytomegalovirus, *EBNA* Epstein–Barr virus nuclear antigen, *EBV* Epstein–Barr virus, *HHV* human herpes virus, *VCA* viral capsid antigen

## Discussion

FT1D is a special type of T1D that was first proposed by the Japanese scholar, Imagawa, in 2000 [1]. FT1D is characterized by rapid onset of DKA within a short time period, with normal to near-normal HbA1c levels at onset and complete β cell destruction [4]. A nationwide survey of FT1D in Japan revealed that it accounts for approximately 20% of cases of T1D [5]. The specific etiology and pathogenesis of FT1D are unclear and may be related to genetic predisposition, autoimmunity, viral infection, and pregnancy.

It is well known that the immunological milieu is significantly changed during pregnancy. However, the issue of whether the clinical and immunogenetic characteristics are different between the cases associated with and without pregnancy remains to be determined.

Although multiple genes have been implicated, HLA class II genes, especially the HLA-DR and DQ genes, are the most important. HLA class II genotypes DRB1*04:05- DQB1*04:01 strongly confer susceptibility to the development of FT1D [6]. In contrast, the frequency of DRB1*09:01-DQB1*03:03 tended to be higher in pregnant women with FT1D than in non-pregnant patients with FT1D [6]. The involvement of HLA class II haplotypes is suggested to vary depending on the presence or absence of pregnancy. In our pregnant patient, the HLA class II haplotypes were *DR8,12/DQ7,4*. To the best of our knowledge, these haplotypes have not been reported as conferring susceptibility to FT1D.

Viral infection is strongly associated with fulminant diabetes, as reported previously, and is evidenced by frequent flu-like symptoms, and it possibly has a seasonal variation in time of onset. Coxsackie B is a group of six types of EVs belonging to the *Picornaviridae* family. Coxsackie B viruses have a tropism for muscle cells and are most common in children but may occur in adults. Clinical manifestations include fever, aseptic meningitis, costochondritis, pharyngitis, myocarditis, diarrhea, and maculopapular non-pruritic rash [7].

Epidemiological studies have indicated an association between EVs and T1D. These viruses have a strong tropism for insulin-producing β cells; the destruction of these cells leads to T1D [8]. The exact mechanisms by which EVs could cause T1D are not known, but direct infection of β cells and virus-induced inflammation may play a role. Some studies have narrowed down the epidemiological association to a subset of EVs: group B Coxsackieviruses [9].

Recent studies have reported a variety of viral infections, including Coxsackievirus B1, B3, B4, A4, A5, A6, CMV, EBV, HHV6, 7, mumps, and parvovirus B19 to be involved in the development of FT1D [10–12]. However, the association between FT1D in pregnancy and certain viral infections has not been reported. Because HLA class II haplotypes with pregnancy and without pregnancy were different, we think it is possible that a different virus causes FT1D. However, our pregnant patient developed a Coxsackievirus B1 infection that manifested as fatigue and was involved in the development of FT1D.

Therefore, this case suggests that Coxsackievirus B infection is associated with FT1D with pregnancy and without pregnancy. DKA is a medical emergency during pregnancy and has high rates of fetal mortality. A prophylactic measure should be available to avoid the development of FT1D in pregnancy. Recent studies have narrowed down the epidemiological association to a subset of EVs: group B Coxsackieviruses; clinical development of a vaccine against T1D-associated EV types has been started [9]. A Coxsackievirus B vaccine may be protective against the development of FT1D in pregnancy.

## Conclusion

We should assess pregnant women with symptoms of both fatigue and acute hyperglycemia for the presence of DKA, even if they are alert when presenting to the hospital. This case highlights that FT1D in pregnancy may be associated with Coxsackievirus B1 infection.

## Data Availability

All data generated or analyzed during this study are included in this published article.

## Abbreviations

CMV: Cytomegalovirus
CPR: C-peptide reactivity
DKA: Diabetic ketoacidosis
EBV: Epstein–Barr virus
EV: Enterovirus
FT1D: Fulminant type 1 diabetes
HbA1c: Glycated hemoglobin
HHV: Human herpes virus
HLA: Human leukocyte antigens
T1D: Type 1 diabetes

## References

[1] Imagawa A, Hanafusa T, Miyagawa J, Matsuzawa Y. A novel subtype of type 1 diabetes mellitus characterized by a rapid onset and an absence of diabetes-related antibodies. Osaka IDDM Study Group. N Engl J Med. 2000;342:301–7. 10.1056/NEJM200002033420501 10655528

[2] Hyöty H, Knip M. Developing a vaccine for type 1 diabetes through targeting enteroviral infections. Expert Rev Vaccines. 2014;13(8):989–99. 10.1586/14760584.2014.933078 24965051

[3] Tsutsumi C, Imagawa A, Ikegami H, Makino H, Kobayashi T, Hanafusa T, *et al.* Class II HLA genotype in fulminant type 1 diabetes: a nationwide survey with reference to glutamic acid decarboxylase antibodies. J Diabetes Investig. 2012;3(1):62–9. 10.1111/j.2040-1124.2011.00139.x PMC4014934 24843547

[4] Hanafusa T, Imagawa A. Fulminant type 1 diabetes: a novel clinical entity requiring special attention by all medical practitioners. Nat Clin Pract Endocrinol Metab. 2007;3:36–45. 10.1038/ncpendmet0351 17179928

[5] Imagawa A, Hanafusa T, Uchigata Y, Kanatsuka A, Kawasaki E, Kobayashi T, *et al.* Fulminant type 1 diabetes nationwide survey in Japan. Diabetes Care. 2003;26:2345–52. 10.2337/diacare.26.8.2345 12882860

[6] Shimizu I, Makino H, Imagawa A, Iwahashi H, Uchigata Y, Kanatsuka A, *et al.* Clinical and immunogenetic characteristics of fulminant type 1 diabetes associated with pregnancy. J Clin Endocrinol Metab. 2006;91:471–6. 10.1210/jc.2005-1943 16278271

[7] Brown EH. Enterovirus infections. Br Med J. 1973;2:169–71. 10.1136/bmj.2.5859.169 PMC1589238 4349337

[8] Hyöty H. Viruses in type 1 diabetes. Pediatr Diabetes. 2016;17(Suppl 22):56–64. J Diabetes Investig 3:62-69, 2012 10.1111/pedi.12370 27411438

[9] Heikki Hyötya B, Leon F, Knipd M. Developing a vaccine for type 1 diabetes by targeting coxsackievirus B. Expert Rev Vaccines. 2018;17(12):1071–83. 10.1080/14760584.2018.1548281 30449209

[10] The committee of Japan Diabetes Society on the research of fulminant type 1 diabetes mellitus. Report of Japan Diabetes Society’s Committee on research on fulminant type 1 diabetes mellitus: analysis of antiviral antibodies at disease onset. J Japan Diab Soc. 2008;51:531–6. (in Japanese, Abstract in English)

[11] Ohara N, Kaneko M, Nishibori T, Sato K, Furukawa T. Fulminant Type 1 Diabetes Mellitus Associated with Coxsackie Virus Type A2 Infection: A Case Report and Literature Review. Intern Med. 2016;55:643–6. 10.2169/internalmedicine.55.5292 26984083

[12] Akatsuka H, Yano Y, Gabazza EC, Morser J, Sasaki R, Suzuki T, *et al.* A case of fulminant type 1 diabetes with Coxsackie B4 virus infection diagnosed by elevated serum levels of neutralizing antibody. Diabetes Res Clin Pract. 2009;84:e50–2. 10.1016/j.diabres.2009.03.009 19362384

